# Supporting Family Carers of Community-Dwelling Elder with Cognitive Decline: A Randomized Controlled Trial

**DOI:** 10.1155/2010/184152

**Published:** 2010-06-10

**Authors:** Birgitte Schoenmakers, Frank Buntinx, Jan Delepeleire

**Affiliations:** Academic Centre of General Practice, Catholic University Leuven, Kapucijnenvoer 33, Blok J, box 7001, 3000 Leuven, Belgium

## Abstract

*Objective*. Caring for a patient with cognitive decline has an important impact on the general well-being of family caregivers. Although highly appreciated, interventions in dementia home care remain mainly ineffective in terms of well-being. Consequently, in spite of an extensive support system, abrupt ending of home care remains more rule than exception. *Method*. The hypothesis was that the intervention of a care counselor, coordinating care in quasi-unstructured way during one year, will alleviate caregivers' feelings of depression. The study population was composed of community-dwelling patients with cognitive decline. A care counselor was at the exclusive disposal of the intervention group. Primary outcome measure was caregiver depression. *Results*. Finally, depression was 6.25 times less frequent in the intervention group. The actual intervention appeared minimal with only ten applications for more support followed by only three interventions effectively carried out. Although caregivers felt burdened and depressed, formal support remained stable. On the other hand, the availability of the care counselor made caregivers feel less depressed with the same amount of support. *Conclusion*. Carers do not always need to be surrounded with more professionals, but they want to feel more supported. In terms of policy, this could have some important implications.

## 1. Introduction

In the last decade, the prevalence of dementia was rapidly growing with a considerable impact on daily live of all involved parties including the broader community [1]. Due to the overcrowd in residential care and to emotional restraints in family carers to have their relative with dementia admitted, dementia patients are most likely to be cared for at home at least for several years. Today, more than 60% of the dementia patients receive home care [1–3]. In Belgium as well as in other western European countries, there is only a restricted admittance to residential care when people suffer from dementia mainly due to the very complex care demands [4]. With an expected increase of dementia prevalence of about 0.5% every five year together with decreasing residential care provisions, family carers will become indispensable partner in dementia care [5] Besides, the economic impact of dementia home care exceeds the total costs of home care of other chronically ill patients [1, 3]. 

Caring for a community-dwelling elder with dementia puts a high burden on family carers [6, 7]. Compared to carers of patients with other chronic diseases, carers of patients with dementia are more often confronted with depressive feelings, experience a higher burden and have been shown to be in a worse general health [8, 9]. The negative impact of the care situation is caused by inefficient coping strategies in carers, rather than by the objective workload [10–13]. However, depressive feelings in the carer are the main direct motive for premature ending of home care, resulting in institutionalization of the dementia patient [14–16]. 

Interventions in dementia home care have been reported as highly appreciated but mainly ineffective [17–19]. On average, carers attach great value to all kinds of support but finally they do not feel less depressed or burdened. Multidisciplinary, hierarchically structured care programs are mostly addressed to the patient without assessing the carers' daily needs [20–22]. As a consequence, in spite of the presence of an extensive or sophisticated support system, an abrupt end to the home care situation remains more rule than exception [15, 23]. In this trial, we tested the hypothesis that the intervention of a care counselor, coordinating home care in a nonhierarchical and quasi-unstructured way during one year, will alleviate carers' feelings of depression.

## 2. Method

### 2.1. Study Question

Will an intervention, consisting in monthly phone calls, a three monthly visit to inventory care needs, organizing formal support and a continuous availability of a care counselor over the course of one year decrease the rate of depression in family carers of community dwelling elder with cognitive decline? 

### 2.2. Study Population and Setting

The study population was recruited among frail community-dwelling elder, characterized by their carers as cognitively impaired. A local home nursing organization, the White-Yellow Cross, listed their elder patients with minimum care dependence (washing, bathing, dressing, transfers and continence) and labeled as cognitively impaired. The care dependence corresponds with at least the lowest home care forfeit reimbursed by the social insurance institute and implies the intervention of a nurse at least once daily. Previous research demonstrated that frailty in older people with a cognitive decline sensitively predicts the presence of a dementia syndrome [1, 24]. Second, the recruitment strategy was justified by the findings that family carers are reliable indicators of a cognitive decline and that the negative impact of home care is evoked by the relative's assumption of a dementia state. [25, 26]. In a later stage, all patients were tested with a Mini Mental State Examinations and those scoring above 22 were excluded. To meet the final inclusion criteria, patients had to be accompanied by a family carer. Exclusion criteria were the absence of minimum care dependence, a severe or terminally ill patient, a definitive institutionalization planned on a short term, no available carer, or an impaired carer. 

The region were recruitment was conducted can be described as a mixed rural-city area of Leuven, Belgium.

### 2.3. Study Design

The intervention study was performed in a controlled design with random assignment to intervention or control group. Randomization for an eighty subjects sample was performed in blocks of ten recruited subjects using the software of www.Randomization.com. 

The elders were consequently visited by the research assistant after their general practitioners had formally been asked permission to visit their patients. The involved general practitioners were formally asked permission for these visits by the trial assistant. Above, general practitioners had first been invited to an information session about the objective of the study in collaboration with the local general practitioners network. Not one general practitioner refused cooperation, mainly due to the absence of a need for active involvement. 

Because study blinding of the subjects in this matter is impossible, the included population was not informed about the exact nature of the trial. Family carers and patients were only asked to participate in interviews with the trial assistant over the course of one year. The trial assistant who did the baseline and outcome interviews and performed all measurements was blinded for the group assignment of the patients.

### 2.4. Intervention

The actual intervention ran from June 2005 until June 2006. The care counselor was a primary care professional with a bachelor degree and was selected on excellence in social and communicative skills and because of her experience in dementia home care. An extra training included a theoretic guidance through local community services addressing dementia home care provided by a skilled general practitioner. Beside, the care counselor was introduced to the home nursing organization, a local service centre for the elder and the local general practitioners network. During the ongoing of the study and in particular with each new intervention, the care counselor was supervised and given feedback by a skilled general practitioner. The care counselor was asked to write down an unstructured report on every provided and extra contact with the carer. 

The care counselor was at the exclusive disposal of the intervention group. Over a course of 12 months, the care counselor guided the family carer in organizing home care. At a first visit, the counselor assisted the family carer in exploring any problematic home care situations. 

Additionally, the care counselor arranged a monthly phone call with the family carer and a three monthly visit. During the intervention period twelve phone calls and four home visits were scheduled. Additionally, the care counselor was within permanent reach for advice by phone, for adjusting home care or for an extra visit. No structured or hierarchical care plan was provided but drawn out following the needs of the family carer and patient. General practitioners were informed about each change in formal or informal home care of their patients. 

Subjects in the control group were not guided or visited by the care counselor but were passively directed to the usual care systems. Both the control group and the intervention group were visited six-monthly by the trial assistant interviewing the family carer and the elder relative. 

For both groups, an accurate inventory of all support installed outside the reach of the trial was reported. Contamination of the effect of the trial intervention by outside support was taken into account in the final analysis.

### 2.5. Outcome Measures and Instruments

Baseline features were recorded after the informed consent and directly following randomization and repeated after six and twelve months; see Table 3. Intervention was initiated after baseline registration. 

Outcome measures and instruments were chosen in accordance with other trials on this topic and based upon the experience with the Qualidem study [1]. Primary outcome measure was defined as depression in the family carer and measured by the Beck Depression Inventory with a score of 10 or more implying mild to moderate depression [27]. Secondary outcome measures were coping behavior, anxiety, and burden in the family carer. 

Burden was measured with the 14 item Zarit Burden inventory [28, 29]. This shortened version of the original Burden Inventory has proved its validity in family caregiving topics. Coping behavior was quantified by the Ways of Coping Checklist [30]. Anxiety was determined by the Trait- subscale of the Stai-instrument [31]. This subscale points out if subjects are prone to anxiety rather than it does reflect a state of mind during a limited period. 

The patient's status was measured with the aid of Frail, the Activities and Instrumental Activities of Daily Living, the Mini Mental State Examination for cognitive status, and the Neuro psychiatric Inventory for behavior [32]. The symptoms described in this instrument were grouped into four categories: psychotic symptoms, disturbing behaviour, mood swings and neurovegetative alterations (sleeping and eating problems, fears). 

Additionally, an extensive quantitative assessment of formal and informal care support was made. 

Finally, for each newly installed care support, the general practitioner was contacted. 

### 2.6. Analysis

The analysis was performed with the SAS version 8.2 software. Sample size calculations were made with a 95% confidence interval and an estimated change in mean depression scores of 2 units. In ANOVA-analysis (one way analysis of variables) a total sample of 46 subjects or 23 in each group was needed to reach a power of 0.9 with a significance level of 0.05. In logistic regression analysis, including two interaction terms, 80 subjects were needed to reach a 0.9 power with a significance level of 0.05. 

The independent variable was the intervention versus usual care. Baseline measures, including the available support, were compared between control and study group by mean differences (expressed in *t*-test) and odds ratios for dichotomized variables. The effect of the intervention was determined by the evolution of depressive scores in the carer with depression (≥10) being the dependent variable. Bivariate analysis was performed to estimate the odds ratio for depression in the intervention versus the control group. In a multiple logistic stepwise regression analysis, the influence of the patient and carer features on the prevalence of depression was calculated. For power reasons, all covariables were sequentially modeled together with the main independent factors. Repeated measures analysis following the hierarchical linear model was used to estimate the mean change differences in depression scores in control and study group over time (longitudinal). The technique used for this analysis allowed the adequate handling of at random missing data and compared both within as between subject changes. All observations of all subjects are used and interpreted based upon likelihood estimation. 

The risk of contamination of the control group was rated unlikely. First, the studied population was not informed about any intervention. Second, general practitioners were only passively involved and were neither aware of the controlled design of the study. They were only contacted when an intervention was proposed to their patient. Third, the local and regional support systems remained stable over time which supported the assumption that minor changes in the usual care did not dramatically influenc the intervention effect. 

### 2.7. Ethical Board

The medical Ethical Board of the Medical School of the Catholic University of Leuven granted formal permission for this trial on 27 January 2005. Permission was given after the reassurance that control and intervention group subjects were not restrained from the usual care systems. Each patient and carer signed a written informed consent at the first visit of the trial assistant. The consent asked permission for participation in a trial studying the care needs of community dwelling elder with cognitive decline and their family carers. The actual participation to an intervention was not mentioned in the consent. The ethical board agreed with this strategy on condition that the involved general practitioner was previously informed about each proposed intervention. If the patient with cognitive decline was not aware of the consequences of study participation, the nearest family member was allowed to sign the informed consent.

## 3. Results

### 3.1. Study Population and Sociodemographic Features (Table 1 and Flow Chart Consort Criteria Shown in Figure 1)

Over a course of 6 months, 346 inhabitants of the target region were eligible for initial screening. All these patients were caredependent, visited daily by a home nurse, and living together with a carer. A preselection was made by the involved home nurse. The drop out (*n* = 49) was mainly due to a normal cognitive status in the patient, recent hospitalization of the patient, the absence of a carer, or an extreme care depending carer. After this stage, 297 were selected for a home visit by the trial assistant. Of these, only 62 patients with their carers were included in the trial. About 20% (*n* = 59) of the dyads refused participation. The most frequent reason of noninclusion was the absence of a carer in home (*n* = 127). Carers not living together with the patient were difficult to reach for the simultaneous interviews. Forty nine carers and patients did not meet other inclusion criteria. Not one of the selected patients had a score higher than 22 on the Mini Mental State Examination. 

Shortly after the inclusion phase, two patients died and one patient was hospitalized for a long term. Baseline measures were available of 59 dyads of carers and patients. Six and twelve months after inclusion 52 and 46 carers and patients were available for the follow-up interviews. Within six months four patients died, one patient and two carers were hospitalized. Within the second half year two additional patients died, one patient and one carer were hospitalized, and two carers refused further participation mainly due to the emotional impact of the interviews. 

Baseline characteristics did not significantly differ between control and study group. In the intervention group, more partners are carers than in the control group. Thanks to the recruitment mechanism all patients were supported by a home nurse. Over half of them were also visited by a home assistant, a cleaning service or a physiotherapist. Most carers rated themselves as being in good health. However, it should be noted that in the intervention group 10 carers were also caredependent. Most carers mentioned that no interdisciplinary contact moment had been organized. 

### 3.2. Baseline Characteristics of Carers and Patients (Table 2)

At baseline, one third of all carers showed at least a mild depression as scored on the BDI-scale (30.5%). The mean score on the depression scale was 8.2. Most carers felt seriously overloaded, with a mean score of 12 on the burden screen. Anxiety scores on the trait-subscale were high in all carers. The carers showed a mix of problem-solving, supporting, and emotional coping behavior. There was no difference in mean scores on these items between the control and intervention group. 

As expected, the patient population was highly care-dependent (see inclusion criteria). Patients showed moderate to high frailty, were limited in performing instrumental tasks of daily living and showed a mild to moderate cognitive decline. Three quarters of the patients were confronted with continence problems. Mood swings and disruptive behavior (screaming, aggressions, etc.) were present in one third of all patients. No differences were noted between control and intervention patients. 

### 3.3. Features of Patient and Carers after 12 Months (Table 4)

One year after the start of the intervention the odds ratio for depression in the treatment arm versus the control group was 0.16 (95% CI 0.03–0.86)**. **Sequentially, taking each carer characteristic and its interaction terms into account, no significant change in the odds ratio for depression was found when gender and having a relationship were introduced. Lower burden levels are followed by a further reduction in depression prevalence in the intervention group as compared to the depression prevalence in the control group (*P* = .06). 

Patient characteristics did not change the odds ratio for depression in a significant way except for neuro-vegetative behavioral disturbances. Intervention group carers confronted with this type of behavior are less prone to depression than their colleagues in the control group. 

### 3.4. Formal Care Support after 12 Months: Usual Care and Proposed Interventions

Besides the regular phone calls and home visits, the care counselor was contacted only once by a carer. All carers rated the phone calls as beneficial. Ten carers applied for extra help. Four of these carers were satisfied with an extra visit of the care counselor. A new intervention was proposed to six carers. In two cases, day care was introduced, two personal alarms, and one offer for extra in-home help were proposed. Three proposals (two alarms, one day-care stay) were effectively carried out. Characteristics of these carers and their relatives were not different from average scores at baseline. 

In both groups, formal care support did not change from baseline to end time, nor was there a difference between intervention and control group (Table 4).

## 4. Discussion

### 4.1. Summary of Main Findings

In this randomized intervention trial, we studied the presence of a care counselor, coordinating home care on the dementia family carers' depression risk rate. At the end of the trial, depression was 6.25 times less frequent in the intervention group carer. The effect of the intervention on the prevalence of depression remained stable when features of patients and carers were taken into account. The recruitment strategy based upon carers' indication of a cognitive decline in their relative combined with a minimum care dependence, proved reliable to detect patients with at least a mild dementia (mean score on MMSE 16/23) [33]. A highly sensitive diagnosis of dementia was not required since the awareness in carers of a cognitive decline of their relative appeared a sufficient criterion to negatively influence the impact of home care [34]. 

The actual intervention of the care counselor was minimal. Ten carers applied for extra support and only one carer called the care counselor outside the provided appointments. Only half of the proposed interventions were effectively carried. Remarkably, it was not the most stressed or loaded carer that applied for an extra intervention. 

Most community-dwelling elders with cognitive decline are cared for by a close female relative (spouse, daughter). The age of the family carer varied from mid forty to above eighty. Carers rate their general health as good although this might be an overestimate due to self-scoring and selection bias. There is a remarkable gap between the healthy and the care-depending carers. Only one carer rated himself in the category “good health but impossible to provide care”. It is likely that carers rate their health as good but that they are reluctant to admit that they cannot offer help. Besides, it should be noted that a small sample of carers was not included at baseline due to health related problems (*n* = 16). 

At baseline, both control and intervention group appeared to be comparable on patient carers and formal support features. One third of the carers seemed at least mildly depressed with a score of ten or more on the BDI. This prevalence corresponds with that reported in international publications. However, the cutoff score of 10 or more which implies mild to moderate depression on the BDI is debatable. Nevertheless, it was not the purpose of this trial to provide a formal diagnosis of depression but to screen carers at risk of it in a sensitive way [35]. The most important disadvantage of the BDI is the score deviation in patients with an important somatic disease [36]. Depression was pointed as primary outcome measure in accordance with other publications in this domain and with conclusions drawn from the preceding population survey [1]. Depression, more than burden or anxiety, reflects the mental wellbeing of the carer and appears to be a sensitive predictor of premature ending of home care [37, 38]. 

Beside a depressive mood, carers experience home care as a heavy burden. It is known from other publications that these feelings of being overburdened are rather unrelated to the objective care burden [39]. The lack of an accessible support system together with inadequate coping strategies and feelings of loneliness, cause carers to feel burdened. Besides, the unpredictable character of dementia and the inherent social isolation make carers very anxious, as reflected in the high scores on the Stai-instrument. To cope with this, carers tend to combine a supporting coping behavior with problem solving behaviour, which is known to be an efficient survival strategy [11]. 

The formal support present at baseline reflects a common dementia home care situation [1]. Due to the recruitment mechanism, all patients were daily visited by a nurse. One carer stopped the home nurse intervention during the trial because of practical reasons. Half of the patients were regularly visited by family help, cleaning service, or physiotherapist. All these services are at least partially reimbursed by the social insurance. The high cost of paid private help, a personal alarm, day care, and meals on wheels are the reasons the absence of these services in common home care situations. Most patients and carers also considered the monthly visit of their general practitioner as organized care management. Basically, very few care plans with multidisciplinary deliberation were set up. General practitioners play their key role in home care and communicate in an unstructured way with other disciplines, mainly when problems occur [40, 41]. 

One year after baseline, in both groups the formal support mechanisms were unchanged. The in-between groups as well as the over-time comparison of this parameter did not reveal any difference. One exception was the item “paid private help” with an increase over time. This observation was due to a new system of reimbursing private home services, introduced by the government. Since no important changes in formal support were noted, a contamination of the trial intervention was not suspected. Beside, interference of support installed independently of the trial was not considered as a disturbing issue. Both groups were free to apply for extra professional help, which was accurately inventoried. 

The care counselor intervened in very few cases. Only half of these interventions were effectively carried out. In spite of high levels of burden, anxiety and depressive feelings, carers appraised the amount of formal support as sufficient. On the other hand, carers remained reluctant to accept another new care provider in their house. Even with the aid of the care counselor, the gap between requesting care support and effectively implementing it in the home care situation still appears to be large. There is some evidence in the literature that carers feel reluctant to appeal for professional help [42, 43]. Besides getting lost in the labyrinth of support systems, carers also experience social pressure to independently fulfill their caring job. Secondly, carers feel shame as well as worries about their cognitively and functionally impaired relative [43].

### 4.2. Comparison with Existing Literature

Although the support strategy reported in our trial is not unknown in geriatric and psychiatric setting, only a very limited amount of trials in dementia research were comparable with the design of our study [44, 45]. Callahan and colleagues proposed the intervention of a care coordinator concentrating on an extended training of patient and carer. In contrast with our study, the control group received augmented usual care. Their findings of improved carer wellbeing were similar to our findings. As compared to the results in our trial, the gains in the carer wellbeing might not justify the socioeconomic costs of training both carer and patient. The trial on befriending with relatives of dementia patients did not redeem the expectation of improved carer wellbeing. It is not unlikely that the high frequency of the “professional friend” visits generated an extra burden in the dementia carer. In contrast with our trial, the amount of visits was reduced to the minimum to avoid the negative feelings related to another professional intruder. Other trials addressing dementia home care related issues by offering a coordinated support used a more hierarchical intervention strategy [46–48]. Carers were expected to run through a fix program integrating different levels of support. Only on indication of the carer or when situations required, the support program was adapted to the actual needs. As appeared in our trial, carers are sufficiently surrounded by professional support. 

### 4.3. Strengths and Limitations of the Study

The strength of the study is the randomized design, with homogenous study groups and the extensive inventory of formal and informal home care in both groups. As compared to other similar trials a major strength is the clean control conditions guaranteed by the subjects' unawareness of an ongoing intervention. In several trials the control group received, due to ethical concerns, an augmented usual care, were put on a waiting list for treatment, or simply received another active intervention [44, 46, 49]. Another important advantage of this support strategy is the low impact on the carers' daily living. There is no burden of attending a training session outdoors and no inconvenient presence of another professional indoors [45, 50]. Previously, it has been demonstrated that carers might lack time and supervising volunteers to organize home care during their absence (“services”). On the other hand, carers report also some extra burden related to the presence of a professional taking over or judging their care-giving tasks [43]. 

A weakness of this study is certainly the small sample size. As known from other publications on this topic, carers often feel too burdened and report lack of the time to participate in a study. This means that the included sample of carers could belong to the best surviving group surrounded by an adequate support system. Nevertheless, prevalence of depression and burden in the carer and the amount of formal support is in accordance with the features of a general sample drawn before in a cross-sectional population survey [1]. Consequently, it is defendable to assume that a representative sample of carers and patients was drawn. Most reports on this research issue deal with a selected study population caused by recruitment via day care centers, local departments of the Alzheimer society, memory clinics, and service centers for elder people [48, 50–53]. A potential bias related to the inclusion of help seeking dyads carer and patient is likely to be present. Evidence exists that this former population is not the most burdened one and handle rather active problem solving strategies [54, 55]. Second, the effect of the intervention is higher than reasonable would be expected. One explanation could be the small sample size. Another plausible explanation could be that the intervention is mainly effective in mildly depressive carers. In case the cutoff point on the depression instrument was lowered to eight, no significant difference was noted between both groups after one year followup. Additionally, the estimated differences of mean depression scores between control and index group appeared not significant. In the range of the baseline moderate or severe depressive carers, no such changes were detected. 

### 4.4. Implications for Future Research and Clinical Practice

Carers do not need to be surrounded with more formal carers, but they want to feel more supported. In terms of policy, this could have some important implications. Instead of inventing new, sophisticated, or complex support mechanisms, home care should become more accessible. A care counselor, familiar with the local care systems, could guide carers through the difficult pathway of home care. In future research, this observation should be repeated in larger groups and over a longer period to assess outcomes such as institutionalization and cost of care. An extended cost analysis should be made to estimate the effect of the intervention on the increasing community costs of dementia home care.

## Figures and Tables

**Figure 1:**
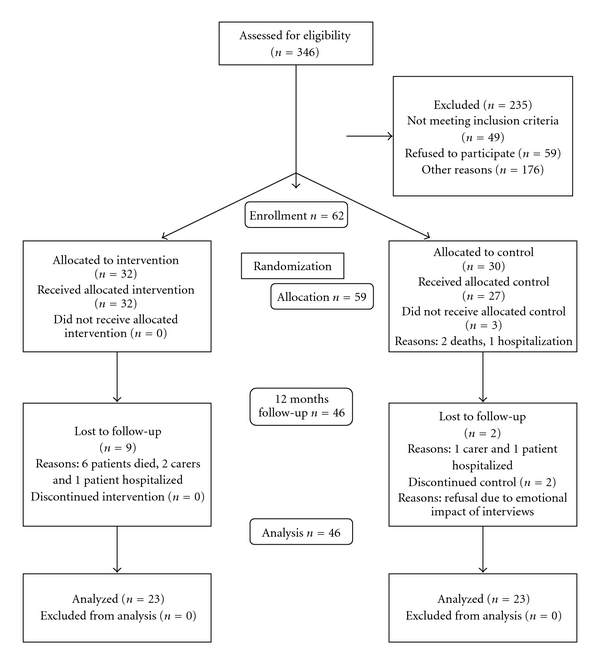
Consort flow chart version 2005.

**Table 1:** Study population at baseline.

Feature	Intervention group *n* = 32	Control group *n* = 27
Carer age	64.4 (SD 12.9)	62.3 (SD 15.1)

Carer gender	7 male	7 male
	25 female	20 female

Carerpatient relation	17 partners	10 partners
	10 sons or daughters	10 sons or daughters
	5 other family	5 other family
		2 no family

Home care		
(i) Home nurse	32	27
(ii) Home assistant	18	12
(iii) Cleaning service	17	7
(iv) Physiotherapist	14	15
(v) Social worker	2	1
(vi) Supervision	8	3
(vii) Meals	4	4
(viii) Personal alarm	5	5
(ix) Day care	3	8
(x) Paid private help	2	0
(xi) Interdisciplinary communication	27 on irregular basis	26 on irregular basis
	1 on regular basis	1 on regular basis
	4 care plan	0 care plan

Health of the carer	22 good health	24 good health
	10 care dependent	1 good but not able to help
		2 care dependent

**Table 2:** Baseline features of patients and carers.

Baseline (cutoff)	Mean scores in all carers (SD)	Prevalence in all carers (dich)	Mean scores and *t*-test intervention versus control group (*P*)	Chi² intervention versus control group (*P*)
*Features carer*				
(i) depression (>9)	8.2 (SD 7.6)		8.5 versus 8.2 (*P* = .9)	
(ii) burden (>9)	11.8 (SD 9.4)		12.9 versus 10.6 (*P* = .4)	
(iii) Anxiety (≥40)	83.6 (SD 14.6)		84 versus 83.3 (*P* = .8)	
(iv) Emotional coping (>21)	17.1 (SD 5.1)		16.9 versus 17.3 (*P* = .7)	
(v) Supporting coping (>20.33)	22.1 (SD 4.5)		21.9 versus 22.4 (*P* = .6)	
(vi) problem solving coping (>27.3)	28.4 (SD 5.0)		28.6 versus 28.3 (*P* = .8)	

*Features patient*				
(i) frailty (≥19)	36.9 (SD 12.8)		38.6 versus 34.9 (*P* = .3)	
(ii) IADL (≥10)	20.5 (SD 4.4)		20.9 versus 19.9 (*P* = .4)	
(iii) MMSE (>23)	16.3 SD (9.6)		17.1 versus 15.2 (*P* = .5)	
(iv) Continence		74.6%		(*P* = .9)
(v) Disruptive behavior		33.90%		(*P* = .02)
(vi) Mood swings		42.37%		(*P* = .2)
(vii) Neurovegetative disturbances		27.12%		(*P* = .4)
(viii) Psychotic features		13.56%		(*P* = .2)

**Table 3:** Features of patient and carer after 12 months.

12 months after baseline (*n* = 46)	Odds ratio for depression in intervention versus control group for each covariable with 95% CI, corrected for gender and relation
*Features carer: *	
Depression	**0.16 (0.03–0.86) **
Co variable	
(i) burden	0.09 (0.007–1.1)
(ii) anxiety	0.3 (0.05–2.3)
(iii) Emotional coping	0.1 (0.01–1.2)
(iv) Supporting coping	0.2 (0.03–1.1)
(v) Problem solving coping	0.2 (0.03–1.6)

*Features of patient*	
(i) Co variable	
(ii) frailty	0.2 (0.3–1.3)
(iii) IADL dependency	0.2 (0.02–1.1)
(iv) Incontinence	0.2 (0.03–1.04)
(v) Disruptive behavior	0.1 (0.03–1.9)
(vi) Mood swings	0.1 (0.01–1.2)
(vii) Neurovegetative disturbances	**0.1 (0.01–0.98)**
(viii) Psychotic features	0.1 (0.01–1.4)

**Table 4:** Formal care support after 12 months.

Type of care	Difference between both groups at 12 months (Chi², *P*)	Overall changes from baseline to 12 months (Chi², *P*)
Home nurse	NA	NA
Home assistant	0.0007 (0.97)	0.2 (0.7)
Cleaning service	0.8 (0.3)	0.04 (0.8)
Physiotherapist	3 (0.08)	0.08 (0.8)
Social worker	0.004 (0.9)	0.04 (0.8)
Supervision	1.2 (0.3)	0.2 (0.6)
Meals	0.3 (0.6)	0.2 (0.6)
Personal alarm	0.3 (0.6)	0.08 (0.8)
Day care	0.7 (0.4)	0.7 (0.4)
Paid private help	2.7 (0.1)	**8.3 (0.004)**
Interdisciplinary communication	1.2 (0.5)	1.3 (0.5)
